# Mitral valve repair versus replacement for endocarditis: A propensity-score matched analysis of early postoperative outcomes

**DOI:** 10.1177/02676591251356417

**Published:** 2025-06-26

**Authors:** Fadi Ibrahim Al-Zubaidi, Umar Shafiq, Harry Smith, Manoraj Navaratnarajah, Maria Pufulete, Gianni D Angelini, Hunaid A Vohra

**Affiliations:** 1 Department of Cardiac Surgery, 156594 Bristol Heart Institute, UK; 2 Department of Cardiac Surgery, University Hospital Southampton, UK; 3 Department of Cardiac Surgery, 156725 Harefield Hospital, UK; 4 Department of Cardiac Surgery, St Thomas’ Hospital, London, UK; 5 Faculty of Health Sciences, 1980 University of Bristol, UK

**Keywords:** mitral valve repair, replacement, endocarditis

## Abstract

**Objective:**

We sought to compare early postoperative outcomes between mitral valve repair (MVr) and replacement (MVR) in patients with mitral valve endocarditis.

**Background:**

The optimal surgical approach for mitral valve endocarditis remains controversial, with some studies suggesting that mitral valve repair may be associated with better outcomes than mitral valve replacement and others suggesting no significant differences. This study compares the early postoperative outcomes between repair and replacement in this cohort using a large national database.

**Methods:**

A retrospective, propensity-score matched analysis was conducted using the national data from the United Kingdom, comparing 1381 patients who underwent repair and 3276 patients who underwent replacement between 2000 and 2019. The primary outcome was in-hospital mortality, and secondary outcomes included prolonged admission (>10 days), re-exploration for bleeding, postoperative stroke, and postoperative dialysis. Binary logistic regression models were conducted in the matched group to further examine the relationship between procedure and outcomes.

**Results:**

After propensity-score matching, 1249 pairs were identified. In-hospital mortality was significantly lower in the repair group (4% vs 7%, *p* < .001). Rates of re-exploration for bleeding (6% vs 9%, *p* = .019), postoperative stroke (1% vs 3%, *p* = .030), and postoperative dialysis (5% vs 7%, *p* = .016) were also significantly lower in the repair group. Binary logistic regression analyses demonstrated repair to be independently associated with lower risk of both mortality (OR:0.65, 95% CI:0.43-0.97, *p* = .034) and re-exploration for bleeding (OR:0.70, 95% CI:0.51-0.96, *p* < .028).

**Conclusions:**

This study suggests that patients receiving repair for mitral valve endocarditis have significantly lower mortality and better early postoperative outcomes than those receiving replacement.

## Introduction

Rates of infective endocarditis in the general population are rising, having increased from 26.6 cases per million in 2009-2010 to 50 cases per million in 2018-19^1^. Infective endocarditis affecting the mitral valve (MV) is a serious condition that can result in significant morbidity and mortality if not promptly and effectively treated. The optimal surgical treatment for patients with this condition remains controversial, with mitral valve repair (MVr) and replacement (MVR) representing the two surgical options. Repair rates for MV endocarditis remain low. A large study in the United States demonstrated a repair rate of 20%.^2^ There are, however, significant variations in practice, with one European centre quoting repair rates of 80%.^3^

Given the relatively low repair rates and therefore, lack of consensus on whether to repair or replace in the context of MV endocarditis, there is a need for further research to better understand the optimal surgical treatment for patients with MV endocarditis. The aim of this study is therefore to compare early postoperative outcomes between MVr and MVR in a large cohort of patients using data from the National Adult Cardiac Surgery Audit (NACSA) database.

## Methods

### Data sources

Our analyses were conducted on the National Adult Cardiac Surgery Audit (NACSA) data, maintained by the National Institute for Cardiovascular Outcomes Research (NICOR). This national database contains clinical information on demographics, pre- and post-operative clinical information including mortality, for all major adult cardiac surgery procedures performed in the United Kingdom. Maintenance and validation are regularly undertaken by the use of reproducible cleaning and maintenance algorithms, with return to individual centres for local validation.^4^ We obtained approval to conduct this study from NICOR and from our institution, the University Hospitals Bristol and Weston National Health Service Foundation Trust Clinical Audit Team, to carry out this research without requiring patient informed consent. The data was received with all identifiable patient information removed; the requirement for patient informed consent was waived and the dataset was adapted for use with statistical analysis software.

### Missingness

The nature of missingness in the dataset was investigated using Little’s test, which returned a significant result (*p* < .001), indicating that data were not completely missing at random. In the following variables, we determined that missingness was likely to be related to the true value (percentage missingness stated in brackets): coronary disease (29%), preoperative AKI (28%), concomitant tricuspid surgery (65%), concomitant coronary artery bypass graft (32%), concomitant ablation (26%), in-hospital mortality (2%), postoperative stroke (12%), postoperative dialysis (11%), re-exploration for bleeding (8%). We therefore imputed missing values with ‘0’ in these variables. In the remaining variables, no attempts were made to replace missing values.

### Ethics statement

The register-based cohort study is part of a research approved by the Health Research Authority (HRA) and Health and Care Research Wales and a need for patients’ consent was waived, as all patients in the database were anonymised (HCRW) (IRAS ID: 257758,23.7.2019).

### Study population

In order to conduct this retrospective study, we obtained data on 4657 patients undergoing mitral valve surgery (MVS) for endocarditis between 2000 and 2019. We included concomitant coronary artery bypass graft (CABG) surgery, concomitant tricuspid valve (TV) surgery and concomitant ablation for atrial fibrillation (AF). We excluded concomitant aortic valve (AV) surgery, as well as patients with missing data for aetiology of MV disease or procedure date.

### Outcomes

The primary outcome was in-hospital mortality. Secondary outcomes included re-exploration for bleeding, prolonged hospital stay (defined as greater than 10 days), post-operative stroke and post-operative dialysis.

### Statistical analysis

Descriptive statistics were used to compare patient characteristics, intraoperative variables and early postoperative outcomes between MVr and MVR in the unmatched and matched cohorts (see Tables 1–4). Continuous variables were reported as means with standard errors, while categorical variables were reported as counts and percentages. Where continuous variables were found to be non-normal, they were reported as medians and interquartile ranges. Chi-squared tests were used to compare categorical variables, and t-tests were used to compare continuous variables. A *p*-value less than 0.05 was considered statistically significant.

**Table 1.** Unmatched cases: univariable comparisons of baseline characteristics.

Variable	Repair (*n* = 1381)	Replace (*n* = 3276)	*p*-value	Missing
Age (years)	58 (±0.4)	58 (±0.3)	0.581	1 (0%)
Male sex	1025 (74%)	962 (71%)	0.011	3 (0%)
BMI	25.0 (±0.17)	25.2 (±0.09)	0.286	157 (3%)
NYHA class III-IV	605 (44%)	1998 (62%)	<0.001	62 (1%)
CCS score			0.025	112 (2%)
I	1113 (82%)	2528 (79%)		
II-III	201 (15%)	518 (16%)		
IV-V	41 (3%)	144 (5%)		
Year of admission			<0.001	54 (1%)
2000-2003	70 (5%)	404 (12%)		
2004-2007	198 (14%)	587 (18%)		
2008-2011	270 (20%)	686 (21%)		
2012-2013	365 (27%)	717 (22%)		
2014-2019	472 (34%)	834 (26%)		
Diabetes	113 (8%)	335 (10%)	0.028	38 (1%)
Hypertension	491 (36%)	1177 (37%)	0.715	64 (1%)
Active smoker	179 (13%)	508 (16%)	0.015	94 (2%)
Preoperative AKI	51 (4%)	352 (11%)	<0.001	1274 (28%)
Urgency			<0.001	5 (0%)
Elective	585 (42%)	593 (18%)		
Urgent	684 (50%)	2005 (61%)		
Emergency	96 (7%)	607 (19%)		
Salvage	14 (1%)	68 (2%)		
Previous MI	55 (4%)	195 (6%)	0.005	72 (2%)
Preoperative COPD	132 (10%)	413 (13%)	0.003	71 (2%)
Previous stroke/TIA	203 (15%)	651 (21%)	<0.001	187 (4%)
PVD	54 (4%)	208 (6%)	0.001	36 (1%)
Preoperative AF	191 (14%)	556 (18%)	0.004	221 (5%)
Coronary disease	189 (18%)	441 (20%)	0.301	1366 (29%)
EF range			<0.001	99 (2%)
Good	667 (49%)	1577 (49%)		
Moderate	270 (20%)	802 (25%)		
Poor	426 (31%)	816 (26%)		
MV lesion			<0.001	135 (3%)
Regurgitation	1339 (99%)	2938 (93%)		
Stenosis	6 (0%)	94 (3%)		
Mixed	11 (1%)	134 (4%)		
Concomitant CABG	167 (12%)	385 (12%)	0.743	1486 (32%)
Concomitant TV surgery	142 (10%)	302 (9%)	0.259	3036 (65%)
Concomitant ablation^1^	25 (13%)	19 (3%)	<0.001	1216 (26%)
CPB time (minutes)	132 (±1.5)	145 (±1.4)	<0.001	183 (4%)
Cross clamp time (minutes)	100 (±1.2)	106.8 (±1.0)	<0.001	206 (4%)

BMI, body mass index; NYHA, New York Heart Association; CCS, Canadian Cardiovascular Society; AKI, acute kidney injury; MI, myocardial infarction; COPD, chronic obstructive pulmonary disease; PVD, peripheral vascular disease; AF, atrial fibrillation; EF, ejection fraction; MV, mitral valve; CABG, coronary artery bypass graft; TV, tricuspid valve, CPB, cardiopulmonary bypass. ^1^Ablation rates calculated in patients with pre-operative AF.

**Table 2.** Matched cases: Univariable comparisons of baseline and intraoperative characteristics.

Variable	Repair (*n* = 1249)	Replace (*n* = 1249)	SMD
Age	58 (±0.43)	59 (±0.41)	0.07
BMI	25 (±0.15)	25 (±0.15)	0.03
Male sex	917 (73%)	873 (70%)	0.08
NYHA class III-IV	601 (48%)	661 (53%)	0.10
CCS score			0.15
I	1012 (81%)	929 (74%)	
II-III	196 (16%)	265 (21%)	
IV-V	41 (3%)	55 (4%)	
Year of admission			0.17
2000-2003	62 (5%)	165 (13%)	
2004-2007	175 (14%)	193 (16%)	
2008-2011	255 (20%)	222 (18%)	
2012-2013	342 (28%)	228 (19%)	
2014-2019	410 (33%)	424 (34%)	
Diabetes	108 (9%)	141 (11%)	0.09
Hypertension	449 (36%)	468 (38%)	0.03
Active smoker	174 (14%)	229 (19%)	0.10
Preoperative AKI	50 (4%)	92 (7%)	0.11
Urgency			0.04
Elective	476 (38%)	459 (37%)	
Urgent	670 (54%)	671 (54%)	
Emergency	88 (7%)	100 (8%)	
Salvage	14 (1%)	17 (1%)	
Previous MI	55 (4%)	75 (6%)	0.07
Preoperative COPD	125 (10%)	172 (14%)	0.10
Previous stroke/TIA	197 (16%)	250 (21%)	0.10
PVD	52 (4%)	86 (7%)	0.11
Preoperative AF	179 (15%)	214 (18%)	0.07
Coronary disease	173 (18%)	171 (19%)	0.02
EF range			0.11
Good	628 (50%)	530 (43%)	
Moderate	246 (20%)	304 (25%)	
Poor	366 (30%)	395 (32%)	
MV lesion			0.08
Regurgitation	1214 (99%)	1085 (91%)	
Stenosis	10 (1%)	69 (6%)	
Mixed	3 (0%)	38 (3%)	
Concomitant CABG	151 (12%)	151 (12%)	0.01
Concomitant TV surgery	127 (10%)	108 (9%)	0.02
Concomitant ablation^1^	23 (13%)	8 (4%)	0.17
CPB Time (minutes)	132 (±1.6)	139 (±1.9)	0.09
Cross clamp time (minutes)	100 (±1.2)	102 (±1.4)	0.02

Key- BMI, body mass index; NYHA, New York Heart Association; CCS, Canadian Cardiovascular Society; AKI, acute kidney injury; MI, myocardial infarction; COPD, chronic obstructive pulmonary disease; PVD, peripheral vascular disease; AF, atrial fibrillation; EF, ejection fraction; MV, mitral valve; CABG, coronary artery bypass graft; TV, tricuspid valve, CPB, cardiopulmonary bypass. ^1^Ablation rates calculated in patients with pre-operative AF.

**Table 3.** Unmatched cohort: univariable comparison of outcome variables.

Variable	Repair (*n* = 1381)	Replace (*n* = 3276)	*p*-value	Missing
Mortality	46 (3%)	346 (11%)	<0.001	71 (2%)
Re-exploration for bleeding	86 (6%)	351 (11%)	<0.001	360 (8%)
Stroke	19 (1%)	127 (4%)	<0.001	565 (12%)
Postoperative dialysis	63 (5%)	366 (11%)	<0.001	527 (11%)
Admitted ≥10 days	611 (49%)	636 (52%)	0.268	59 (1%)
Length of stay (days)	9 (7-15)	10 (7-17)	0.022	59 (1%)

**Table 4.** Univariable outcomes in matched cohort.

Variable	Repair (*n* = 1249)	Replace (*n* = 1249)	*p*-value
Mortality	44 (4%)	90 (7%)	<0.001
Re-exploration for bleeding	79 (6%)	110 (9%)	0.019
Stroke	17 (1%)	32 (3%)	0.030
Postoperative dialysis	62 (5%)	91 (7%)	0.016
Admitted ≥10 days	611 (49%)	636 (52%)	0.268
Length of stay (days)	10 (7-16)	10 (7–17)	0.317

To minimize selection bias, a propensity-score matching analysis was planned. Propensity-score matching was performed using the ‘MatchIt’ package in R Core Team (2021). R: A Language and Environment for Statistical Computing. R Foundation for Statistical Computing, Vienna, Austria. Version 4.0.5. All descriptive and regression analyses were performed using SPSS version 26.0 (IBM Corp., Armonk, NY, USA).

Propensity scores were calculated using a logistic regression model that included the following covariates: sex, New York Heart Association (NYHA) class, Canadian Cardiovascular Society (CCS) score, diabetes, hypertension, smoking status, preoperative acute kidney injury (AKI), urgency, previous myocardial infarction (MI), previous chronic obstructive pulmonary disease (COPD), previous stroke or transient ischaemic attack (TIA), peripheral vascular disease (PVD), preoperative AF, coronary artery disease, ejection fraction (EF) range, MV lesion, body mass index (BMI) and age. Patients in the MVr group were matched to patients in the MVR group using a nearest-neighbour matching algorithm in a 1:1 ratio, based on the logit of the propensity score using calipers of width equal to 0.2 of the standard deviation of the logit of the propensity score.

Binary logistic regression models were used to examine the association between procedure type and outcomes in the overall matched cohort (see Table 5). We then conducted separate regression models for mortality in the matched MVr and MVR groups (see Table 6), to determine whether predictors of mortality differed between the two (see Table 6). We have reported odds ratios and 95% confidence intervals for all covariates included in the models.

**Table 5.** Multivariable outcomes in matched cohort.

Variable	Outcome		
	Mortality	Re-exploration	Admission ≥10 days
Mitral repair	0.65 (0.43-0.97) *p* = .034	0.70 (0.51-0.96) *p* = .028	0.96 (0.81-1.14) *p* = .636
Age	1.02 (1.00-1.03) *p* = .018	0.91 (0.32-2.54) *p* = .851	0.41 (0.21-0.80) *p* = .009
Male sex	0.69 (0.46-1.03) *p* = .069	1.05 (0.75-1.47) *p* = .790	0.99 (0.83-1.18) *p* = .905
NYHA class III-IV	2.01 (1.31-3.11) *p* = .002	1.20 (0.87-1.65) *p* = .266	1.11 (0.94-1.31) *p* = .219
CCS score			
0 (reference)			
I-II	0.84 (0.50-1.41) *p* = .506	1.11 (0.75-1.63) *p* = .599	1.06 (0.86-1.31) *p* = .598
III-IV	1.92 (0.92-4.00) *p* = .081	1.69 (0.87-3.25) *p* = .119	1.17 (0.75-1.82) *p* = .484
Admission year			
2000-2003 (reference)	0.99 (0.51-1.92) *p* = .972	2.13 (1.04-4.37) *p* = .040	0.71 (0.50-0.99) *p* = .046
2004-2007	0.78 (0.41-1.51) *p* = .461	2.18 (1.08-4.39) *p* = .030	0.63 (0.45-0.87) *p* = .005
2008-2011	0.52 (0.25-1.08) *p* = .078	1.96 (0.96-3.99) *p* = .065	0.74 (0.53-1.02) *p* = .064
2012-2015	0.18 (0.08-0.39) *p* < .001	2.06 (0.89-4.75) *p* = .092	1.17 (0.78-1.76) *p* = .450
2016-2019			
Diabetes	2.13 (1.30-3.48) *p* = .003	1.17 (0.74-1.85) *p* = .515	1.50 (1.14-1.98) *p* = .004
Active smoker	1.00 (0.57-1.76) *p* = .989	0.78 (0.49-1.25) *p* = .304	1.13 (0.89-1.43) *p* = .306
Preoperative AKI	3.22 (1.91-5.45) *p* < .001	1.89 (1.09-3.29) *p* = .023	1.12 (0.78-1.61) *p* = .536
Previous MI	1.08 (0.52-2.22) *p* = .841	1.26 (0.68-2.31) *p* = .466	1.14 (0.78-1.66) *p* = .507
Previous COPD	1.35 (0.83-2.22) *p* = .230	1.33 (0.87-2.04) *p* = .191	1.07 (0.83-1.38) *p* = .603
Previous stroke/TIA	1.40 (0.09-2.20) *p* = .139	1.30 (0.90-1.88) *p* = .160	1.12 (0.91-1.39) *p* = .280
PVD	2.21 (1.22-4.00) *p* = .009	0.96 (0.51-1.80) *p* = .898	0.82 (0.57-1.17) *p* = .278
Preoperative AF	1.17 (0.73-1.85) *p* = .517	0.89 (0.58-1.36) *p* = .588	0.88 (0.70-1.11) *p* = .292
EF range			
Good (reference)			
Moderate	1.58 (0.97-2.58) *p* = .066	0.78 (0.51-1.19) *p* = .246	1.16 (0.93-1.46) *p* = .192
Poor	4.71 (2.48-8.97) *p* < .001	0.82 (0.44-1.54) *p* = .542	0.76 (0.54-1.08) *p* = .131
Mitral stenosis	0.64 (0.26-1.54) *p* = .314	0.64 (0.30-1.39) *p* = .263	0.58 (0.35-0.94) *p* = .029

NYHA, New York Heart Association; CCS, Canadian Cardiovascular Society; AKI, acute kidney injury; MI, myocardial infarction; COPD, chronic obstructive pulmonary disease; TIA, transient ischaemic attack; PVD, peripheral vascular disease; Preoperative AF, atrial fibrillation; EF, ejection fraction.

**Table 6.** Multivariable associations with mortality in MVr and MVR (matched cohorts).

Variable	Cohort	
	Repair (*n* = 1249)	Replace (*n* = 1249)
Age	1.03 (1.00-1.06) *p* = .038	1.01 (0.99-1.03) *p* = .194
Male sex	0.44 (0.22-0.90) *p* = .023	0.82 (0.50-1.35) *p* = .437
NYHA class III-IV	2.18 (1.04-4.58) *p* = .039	1.88 (1.09-3.25) *p* = .023
CCS score		
0 (reference)		
I-II	0.49 (0.16-1.48) *p* = .204	1.08 (0.59-1.98) *p* = .797
III-IV	2.48 (0.73-8.42) *p* = .144	1.97 (0.75-5.22) *p* = .171
Admission year		
2000-2003 (reference)		
2004-2007	0.59 (0.17-2.05) *p* = .406	1.01 (0.45-2.24) *p* = .985
2008-2011	0.48 (0.15-1.58) *p* = .227	0.82 (0.37-1.83) *p* = .625
2012-2015	0.13 (0.03-0.57) *p* = .007	0.88 (0.38-2.06) *p* = .774
2016-2019	0.15 (0.04-0.60) *p* = .008	0.16 (0.06-0.41) *p* < .001
Diabetes	2.01 (0.84-4.79) *p* = .116	2.24 (1.20-4.16) *p* = .011
Active smoker	1.23 (0.43-3.48) *p* = .703	0.96 (0.49-1.87) *p* = .895
Preoperative AKI	3.31 (1.23-8.88) *p* = .018	3.42 (1.77-6.62) *p* < .001
Previous MI	1.15 (0.33-3.99) *p* = .824	0.91 (0.36-2.31) *p* = .838
Previous COPD	0.90 (0.34-2.40) *p* = .836	1.65 (0.91-2.98) *p* = .098
Previous stroke/TIA	1.61 (0.73-3.56) *p* = .242	1.38 (0.79-2.41) *p* = .260
PVD	2.70 (0.93-7.81) *p* = .067	1.93 (0.92-4.05) *p* = .083
Preoperative AF	1.15 (0.51-2.59) *p* = .738	1.16 (0.65-2.07) *p* = .608
EF range		
Good (reference)		
Moderate	1.94 (0.83-4.51) *p* = .123	1.40 (0.75-2.62) *p* = .291
Poor	3.24 (0.98-10.73) *p* = .055	5.92 (2.72-12.9) *p* < .001
Mitral stenosis	2.15 (0.23-19.87) *p* = .499	0.43 (0.16-1.14) *p* = .091

Key- NYHA, New York Heart Association; CCS, Canadian Cardiovascular Society; AKI, acute kidney injury; MI, myocardial infarction; COPD, chronic obstructive pulmonary disease; TIA, transient ischaemic attack; PVD, peripheral vascular disease; Preoperative AF, atrial fibrillation; EF, ejection fraction.

## Results

### Unmatched cohort

In terms of baseline characteristics and intraoperative variables, we observed significant differences between the unmatched MVr (*n* = 1381) and MVR (*n* = 3276) groups. Patients undergoing MVr were more likely to be male (74% vs 71%, *p* = .011). They were less likely to present with NYHA class III-IV symptoms (44% vs 62%, *p* < .001) and had lower Canadian Cardiovascular Society (CCS) scores (*p* = .025). MVr patients also had lower rates of preoperative AKI (4% vs 11%, *p* < .001), previous stroke/TIA (15% vs 21%, *p* < .001) and preoperative AF (14% vs 18%, *p* = .004). They were more likely to undergo concomitant AF ablation (13% vs 3%, *p* < .001), had shorter cardiopulmonary bypass (CPB) times (132 minutes vs 145 minutes, *p* < .001) and shorter aortic cross-clamp (AoX) times (100 minutes vs 107 minutes, *p* < .001) (see Table 1).

There were statistically significant differences in early post-operative outcomes in the unmatched cohorts. Patients undergoing MVr had lower rates of mortality (3% vs 11%, *p* < .001), re-exploration for bleeding (6% vs 11%, *p* < .001), stroke (1% vs 4%, *p* < .001) and post-operative dialysis (5% vs 11%, *p* < .001). Patients undergoing MVr also had shorter overall hospital stays (9 days vs 10 days, *p* < .001), however there were no significant differences in rates of patients staying in hospital beyond 10 days (49% vs 52%, *p* = .022).

### Matched cohort

Propensity-score matching yielded 1249 pairs of matched MVr and MVR cases (see Table 2). There were no significant differences between the two groups for most variables, as standardized mean differences (SMD) were less than 0.10 for all but five variables (see Table 2). The variables that differed between the two groups despite matching were CCS scores (SMD = 0.15), year of admission (SMD = 0.17), preoperative AKI (SMD = 0.11), PVD (SMD = 0.11) and EF range (SMD = 0.11).

After propensity-score matching, patients undergoing MVr had significantly lower rates of mortality (4% vs 7%, *p* < .001), re-exploration for bleeding (6% vs 9%, *p* = .019), stroke (1% vs 3%, *p* = .030), and postoperative dialysis (5% vs 7%, *p* = .016) (see Table 4). There were no significant differences in the proportion of patients admitted for greater than 10 or the length of stay between the two groups.

### Multivariable regression analysis

Binary logistic regression in the matched cohort revealed MVr to be independently associated with a lower risk of mortality (OR:0.65, 95%CI:0.43-0.97, *p* = .034) or re-exploration for bleeding (OR:0.70, 95%CI:0.51-0.96, *p* = .028). Poor EF had the strongest independent association with mortality (OR:4.71, 95%CI:2.48-8.97, *p* < .001). Other independent predictors of mortality included preoperative AKI (OR:3.22, 95%CI:1.91-5.45, *p* < .001), NYHA class III-IV (OR:2.01, 95%CI:1.31-3.11, *p* = .002), diabetes (OR:2.13, 95%CI:1.30-3.48, *p* = .003), PVD (OR: 2.21, 95%CI:1.22-4.00, *p* = .009) and advancing age (OR:1.02, 95%CI:1.00-1.03, *p* = .018) (see Table 5). In terms of re-exploration for bleeding, preoperative AKI was an independent predictor (OR:1.89, 95%CI:1.09-3.29, *p* = .023). Diabetes was the only independent predictor for hospital stay greater than 10 days (OR:1.50, 95%CI:1.14-1.98, *p* = .004). Advancing age (OR:0.41, 95%CI:0.21-0.80, *p* = .009) and mitral stenosis (OR:0.58, 95%CI:0.35-0.94, *p* = .029) were found to be protective factors.

Separate modelling demonstrated common and different predictors of early mortality in MVr and MVR. In patients undergoing repair, advancing age (OR:1.03, 95%CI:1.00-1.06, *p* = .038), NYHA class III-IV (OR:2.18, 95%CI:1.04-4.58, *p* = .039) and preoperative AKI (OR:3.31, 95%CI:1.23-8.88, *p* = .018) were independent predictors for mortality, whilst male sex was a protective factor (OR:0.44, 95%CI:0.22-0.90, *p* = .023). In patients undergoing replacement, NYHA class III-IV (OR:1.88, 95%CI:1.09-3.25, *p* = .023), diabetes (OR:2.24, 95%CI:1.20-4.16, *p* = .011), preoperative AKI (OR:3.42, 95%CI:1.77-6.62, *p* < .001) and poor LVEF (OR:5.92, 95%CI:2.71-12.9, *p* < .001) were independent predictors of mortality. There was no relationship between sex and mortality in MVR.

## Discussion

**Figure 1.**
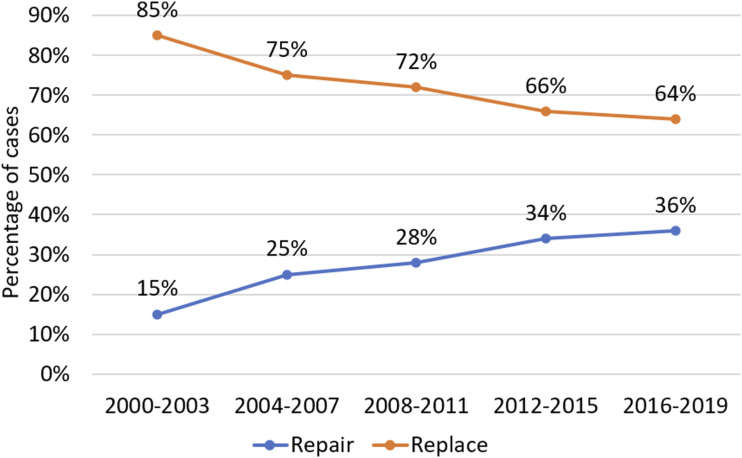
Trends in repair rates for mitral valve endocarditis in overall cohort.

**Figure 2.**
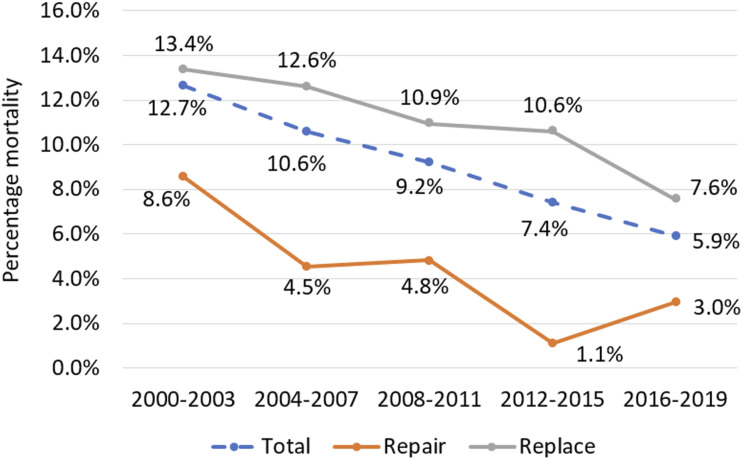
Trends in mortality by procedure: total, mitral repair and mitral replacement.

**Figure 3.**
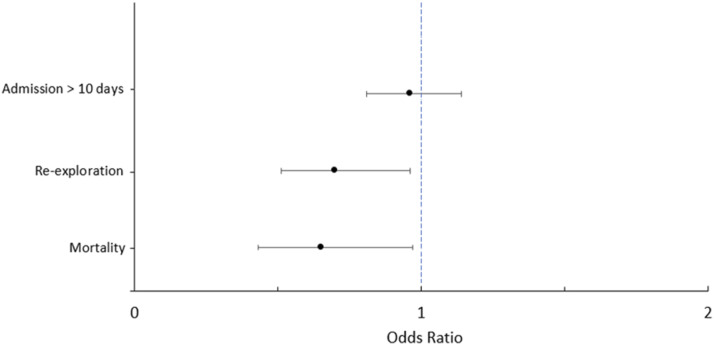
Forest plot demonstrating the independent relationship between mitral repair and outcomes in matched population. Odds ratios ±95% confidence intervals.

There are significant differences between patients with MV endocarditis receiving MVr and MVR. Our analysis reveals patients receiving MVr to be significantly less comorbid than those undergoing MVR. They had shorter CPB and AoX times, shorter hospital stays and superior postoperative outcomes. After propensity-score matching, these significant outcome differences persist, with MVr resulting in reduced mortality, re-exploration for bleeding, stroke and postoperative dialysis compared to MVR. Regression analysis in the matched cohort was designed to minimise the impact of confounding variables. Despite this, MVr emerged as independently associated with a lower risk of both mortality and re-exploration for bleeding. Whilst this is encouraging data supporting the repair of mitral valve endocarditis where possible, this may also relate to unmeasured risk factors associated with patients receiving a replacement. Trends in MV surgery for endocarditis shifted significantly in the UK between 2000 and 2019. Repair rates have risen consistently from 15% in 2000-2003 to 36% in 2016-2019 (see Figure 1). Furthermore, early postoperative mortality rates have dropped significantly, from 8.6% to 3.0% post-repair and 13.4% to 7.6% post-replacement (see Figures 2 and 3).

Our findings show that UK practice is steadily shifting and embracing evidence in the literature which we have corroborated with our results, demonstrating the superiority of MVr over MVR where appropriate. In terms of early outcomes, our results support a repair-orientated approach for MV endocarditis. This confirms the findings of a range of smaller studies which yielded similar results,^3,5–8^ A pertinent follow-on question is, in which patients should we seek to perform repair? Clearly, the extent of infection is one of the most important considerations. We were unable to elucidate further on this given the nature of national registry data, however Omoto et al suggested based on findings in their paper that the extent of the infected lesion is the key variable when deciding whether to repair or replace.^8^ Liang et al demonstrated the importance of early intervention, showing that early surgery was associated with better short and long-term postoperative outcomes.^9^ In a large systematic review and meta-analysis, Narayanan et al. looked at over 21 studies and also concluded that early intervention is particularly crucial in reducing postoperative mortality in patients with endocarditis.^10^ Furthermore, El Khoury’s group have repeatedly demonstrated that it is possible to achieve repair rates of 80% for endocarditis when an aggressive repair-orientated, early intervention approach is taken.^3,11–13^ Both patch and non-patch repair techniques were demonstrated to result in safe, durable results compared to MVR.

Interestingly, when comparing our separate multivariable mortality models for mortality in the MVr and MVR cohorts (see Table 6), age emerged as an independent predictor of mortality in patients undergoing MVr but not in those undergoing MVR. The odds ratio suggests a 3% increased mortality risk associated with each additional year in age, which translates to a 30% increased risk of mortality per decade. This is a clinically significant difference and suggests that we should take a cautious approach to repairing MV in the context of elderly patients with endocarditis. This may be caused by poorer native tissue quality being less amenable to support a repair following the damage caused by endocarditis. Other important predictors of poor outcomes include NYHA class III-IV, preoperative AKI, preoperative diabetes and poor LVEF. Patients fulfilling these criteria should be optimized as much as possible prior to intervention. Recent surgery (2016-2019) emerged as an independent protective factor against mortality, suggesting improving surgical practices per se have reduced mortality rates over time. This is encouraging and, in line with univariable trends, confirms that the UK is heading in the right direction in terms of reducing mortality following surgery for MV endocarditis.

The uniquely large UK sample size based on nationally collected data provides strength to our study. Multivariable analysis allowed us to account for the impact of covariates on observed outcomes. It is important to acknowledge the limitations of our study design, which must be taken into account when drawing conclusions from our findings. Whilst the NICOR database undergoes regular maintenance and validation, given its size we cannot rule out the possibility of some data being recorded incorrectly. There are also areas in our analysis where a lack of granularity in the data limited the conclusions we were able to make; data on the different types of repairs performed, as well as information on why surgeons chose not to repair in MVR cases, would have allowed us to comment further on the reasons behind the observed outcome differences. Crucially, lack of long-term follow up data limited out study to looking at early post-operative outcomes. Given that we made no attempt to systematically replace all missing data, our multivariable analyses were conducted as complete-case analyses. The size of the dataset and the relatively low levels of missingness in the data limit the impact of these factors. Whilst recognising these limitations, our study confirms that there is indeed a relationship between MV procedure and post-operative outcomes for patients with MV endocarditis.

## Conclusions

(1)- Early postoperative outcomes are superior post MVr for endocarditis.
(2)- Age is a poor prognostic factor for repairing MV endocarditis.
(3)- Overall mitral repair rate for endocarditis in the UK has increased over time.
(4)- Early surgery and repair-orientated strategy combined yield high repair rates and good postoperative outcomes.

## Abbreviations

MV: Mitral valve
MVr: Mitral valve repair
MVR: Mitral valve replacement
NACSA: National Adult Cardiac Surgery Audit
NICOR: National Institute for Cardiovascular Outcomes Research
HRA: Health research authority
HCRW: Health and Care Research Wales
MVS: Mitral valve surgery
CABG: Coronary artery bypass graft
TV: Tricuspid valve
AF: Atrial fibrillation
AV: Aortic valve
NYHA: New York Heart Association
CCS: Canadian Cardiovascular Society
AKI: Acute kidney injury
MI: Myocardial infarction
TIA: Transient ischaemic attack
PVD: Peripheral vascular disease
EF: Ejection fraction
CPB: Cardiopulmonary bypass
AoX: Aortic cross-clamp
SMD: Standardized mean difference

## References

[1] Thornhill MH Dayer MJ Nicholl J , et al. An alarming rise in incidence of infective endocarditis in England since 2009: why? Lancet 2020; 395: 1325–1327. 32334690 10.1016/S0140-6736(20)30530-4

[2] Toyoda N Itagaki S Egorova NN , et al. Real-world outcomes of surgery for native mitral valve endocarditis. J Thorac Cardiovasc Surg 2017; 154: 1906–1912. 28942975 10.1016/j.jtcvs.2017.07.077

[3] Solari S Navarra E de Kerchove L , et al. Mitral valve repair for endocarditis. J Card Surg 2022; 37: 4097–4102. 34390270 10.1111/jocs.15891

[4] Hickey G Grant SW Cosgriff R , et al. Clinical registries: governance, management, analysis and applications. Eur J Cardio Thorac Surg 2013; 44: 605–614. 10.1093/ejcts/ezt018 23371972

[5] Oliver L Leauthier M Jamme M , et al. Mitral valve repair is better than mitral valve replacement in native mitral valve endocarditis: results from a prospective matched cohort. Arch Cardiovasc Dis 2022; 115: 160–168. 35249849 10.1016/j.acvd.2022.02.002

[6] Helmers MR Fowler C Kim ST , et al. Repair of isolated native mitral valve endocarditis: a propensity matched study. Semin Thorac Cardiovasc Surg 2022; 34: 490–499. 34197918 10.1053/j.semtcvs.2021.05.025

[7] Kanemitsu H Nakamura K Fukunaga N , et al. Long-term outcomes of mitral valve repair for active endocarditis. Circ J 2016; 80: 1148–1152. 27039946 10.1253/circj.CJ-15-1062

[8] Omoto T Aoki A Maruta K , et al. Operative timing and feasibility of mitral valve repair in active infective endocarditis. Ann Thorac Cardiovasc Surg 2023; 29: 23–28. 36328571 10.5761/atcs.oa.22-00135 PMC9939675

[9] Liang F Song B Liu R , et al. Optimal timing for early surgery in infective endocarditis: a meta-analysis. Interact Cardiovasc Thorac Surg 2016; 22: 336–345. 26678152 10.1093/icvts/ivv368 PMC4986570

[10] Anantha Narayanan M Mahfood Haddad T Kalil AC , et al. Early versus late surgical intervention or medical management for infective endocarditis: a systematic review and meta-analysis. Heart 2016; 102: 950–957. 26869640 10.1136/heartjnl-2015-308589

[11] de Kerchove L Price J Tamer S , et al. Extending the scope of mitral valve repair in active endocarditis. J Thorac Cardiovasc Surg 2012; 143: S91–95. 22306214 10.1016/j.jtcvs.2012.01.049

[12] Solari S De Kerchove L Tamer S , et al. Active infective mitral valve endocarditis: is a repair-oriented surgery safe and durable? Eur J Cardio Thorac Surg 2019; 55: 256–262. 10.1093/ejcts/ezy242 30085002

[13] Solari S Navarra E de Kerchove L , et al. Mitral valve repair for endocarditis. J Card Surg 2022; 37: 4097–4102. 34390270 10.1111/jocs.15891

